# Lifetime prevalence of mental disorders among first and second generation individuals with Turkish migration backgrounds in Germany

**DOI:** 10.1186/s12888-017-1333-z

**Published:** 2017-05-11

**Authors:** Demet Dingoyan, Holger Schulz, Ulrike Kluge, Simone Penka, Azra Vardar, Alessa von Wolff, Jens Strehle, Hans-Ulrich Wittchen, Uwe Koch, Andreas Heinz, Mike Mösko

**Affiliations:** 1 Department of Medical Psychology, Study group on Psychosocial Migration Research, University Medical Center Hamburg-Eppendorf, Martinistraße 52, Building W(est)26, 20246 Hamburg, Germany; 2 Clinic for Psychiatry and Psychotherapy, Charité – University Medicine Berlin, Campus Mitte, Berlin, Germany; 3 Berlin Institute for Integration and Migration Research, Department Migration, Mental and Physical Health and Health Promotion, Faculty of Humanities and Social Sciences, Humboldt University Berlin, Berlin, Germany; 4 Institute of Clinical Psychology and Psychotherapy, Technische Universität Dresden, Dresden, Germany

**Keywords:** Mental disorder, Prevalence, Migration, Turkish, Composite International Diagnostic Interview (CIDI)

## Abstract

**Background:**

This paper focuses on the lifetime prevalence of mental disorders in individuals with Turkish migration backgrounds in Germany, as there is a lack of reliable epidemiological data on this subject.

**Methods:**

In total, 662 adults with Turkish migration backgrounds were interviewed in Hamburg and Berlin by trained, bilingual interviewers using the computerized Composite International Diagnostic Interview (CIDI DIA-X Version 2.8) to assess diagnoses according to the DSM-IVTR.

**Results:**

The analyses showed a weighted lifetime prevalence of 78.8% for any mental disorder, 21.6% for more than one and 7.3% for five or more disorders. Any mood disorder (41.9%), any anxiety disorder (35.7%) and any somatoform disorder/syndrome (33.7%) had the highest prevalences. Despite the sociodemographic differences between the first and second generations, there were no significant differences in the lifetime prevalence between generations, with the exception of any bipolar disorder. Female gender, older age and no current partnership were significantly associated with the occurrence of any mood disorder.

**Conclusions:**

Overall, the results indicate a high lifetime prevalence in individuals with Turkish migration backgrounds in Germany. These initial data are highly relevant to the German clinical and psychosocial healthcare system; however, the methodological limitations and potential biases should be considered when interpreting the results.

## Background

Migration is a universal phenomenon, which includes highly heterogeneous processes and a series of events with long-term consequences for migrants [1]. Within the international research context the term migrant is defined as ‘a person who is living in a country other than his or her country of birth’ [2]. Migration is often considered a critical life event promoting the development of physical and mental diseases in individuals due to the increased adaptation and coping expectations [1, 3–5]. However, studies indicate a health advantage among different migrant groups and ethnic minorities (healthy-migrant effect) that diminishes with increased residence time and across generations [6–9]. When considering generational differences, meta-analyses not only yielded higher prevalence and incidence rates of schizophrenia and other psychotic disorders in first generation migrants but also increased levels within the second generation [10, 11]. In contrast, a meta-analysis by Swinnen and Selten (2007) [12] showed only a slightly increased risk of depressive disorders in first generation migrants and their descendants (1.38, 95%, CI: 1.17–1.62). A systematic review by Lindert et al. (2009) [13] also could not determine significant divergences in the lifetime prevalences of depression and anxiety disorders between labor migrants (depression: 20%, anxiety disorders 21%) and the US general population (depression: 22%, anxiety disorders: 18%). However, the prevalence rates in refugees, which were considered separately, were twice as high as those in the labor migrants (depression: 44%, anxiety disorders: 40%). In this respect, it should be mentioned that the review is limited by the prevalence rates of the individual included studies, which varied greatly (depression: 3 ± 81%, anxiety disorders: 5 ± 90%) presumably due to the various measurement methods and recruitment strategies.

Regarding individual groups, extensive national health surveys in the US suggest lower lifetime prevalence rates of any depressive disorder, any anxiety disorder and any mental disorder in various migrant groups in comparison to their host society, i.e., in Latinos vs Non-Latinos [14], in Mexican Americans vs Non-Hispanic Whites [15], and in Hispanics and Non-Hispanic Blacks vs Non-Hispanic Whites [16]. Furthermore, several studies have reported the following findings: (1) an increased lifetime prevalence of mental disorders in descendants compared to the first migrant generation [14–20]; (2) a negative association between age at migration and the development of mental disorders [17, 18, 20–23]; and (3) a positive association between the duration of residence and an increased risk of disorder development [18, 20, 22, 23].

A cross-national household survey that included 11 European countries stated that older (age ≥ 55 years) first generation migrants (i.e. individuals who were born outside their current country of residence) living in Northern and Western Europe had a moderately increased risk of depression (1.42, 95% CI, 1.28–1.59) compared to non-migrants [24]. A population-based health survey in Belgium also suggests an increased prevalence rate of depressive symptoms and generalized anxiety disorders in Turkish and Moroccan first generation migrants (i.e. individuals with current foreign nationality and foreign country of birth) when compared to EU-member migrants [25, 26]. Increased prevalence rates of depressive and anxiety disorders were also found in the Netherlands in first generation Turkish and Moroccan migrants (i.e. individuals with Turkish or Moroccan country of birth) compared to the general population [27–29].

At this point in time, Germany has no equivalent studies on the lifetime prevalences of mental disorders in individuals with Turkish migration backgrounds. Individual clinical and health care studies found a higher mental strain in Turkish patient groups (i.e. foreign country of birth of the individuals themselves or of at least one of the parents or the foreign first language of the individuals) [30, 31] and in women with Turkish migration backgrounds, especially within the second generation (i.e. first generation: individuals who migrated in the framework of German-Turkish recruitment agreement or a family unification when older than 15 years, second generation: individuals with at least one Turkish parents who were born and/or raised in Germany) [32, 33].

## Aims

Approximately 3 million individuals with a Turkish migration background live in Germany, comprising the largest migrant group in Germany [34]. Taking an inductive approach, this paper will report on (a) the sociodemographic characteristics of individuals with Turkish migration backgrounds considering generational status; (b) the lifetime prevalence of mental disorders (DSM-IV/ICD-10) and comorbidity, considering possible generation effects, and (c) the sociodemographic predictors of the five main diagnosis groups (any mood disorder, any anxiety disorder, any eating disorder, any substance use disorder, any somatoform disorder/syndrome).

## Methods

Within the context of a research project funded by the Volkswagen Foundation, data on the prevalence of mental disorders in individuals with Turkish migration backgrounds were collected. The research process was approved by the ethics committees of the Hamburg chamber of psychotherapists and the ethics commission and data commissioner of the Charité University Medicine Berlin. The full study protocol including the study design and the data collection process has been published previously [35] but is briefly summarized as follows.

### Sample

The study was administered from August 2011 to July 2012. The respondents were adults (ages 18–65 years) with a Turkish migration background living in Hamburg and Berlin (Germany). The inclusion criteria comprised participants (1) having a Turkish migration background based on the definition of the German Federal Statistical Office [34], (2) being older than 18 and younger than 65 years at the time of data collection, (3) having sufficient mobility (as no house visits were made), and (4) signing the consent form. Individuals were considered as having a Turkish migration background if they moved to the German Federal Republic after 1949, were Turkish nationals born in Germany, or were German-born citizens with at least one parent who was a migrant from Turkey or a Turkish national. The total sample of completed interviews, *n* = 662 (Hamburg: *n* = 376, Berlin *n* = 286), was stratified according to gender, age and education based on micro-census data (personal communication with regional statistical office, 2012).

### Data collection

In *Hamburg,* a random sample of 19 districts (with a high quantity and density of residents of Turkish citizenship) was selected through the regional population register. The sample consisted of persons with Turkish citizenship (*N* = 7239) and persons with Turkish migration backgrounds (*N* = 3098) who had German citizenship (i.e., because of naturalization). The latter sample group was identified through the onomastic procedure (a proven name algorithm by Humpert & Schneiderheinze, 2000 [36]). These individuals were contacted by letter, through random selection, and asked for their voluntary participation in the study. Due to a low response rate (average 2.5%), additional snowball sampling was applied in the final 3 months of the data collection, in which participants were recruited (*N* = 320) through cultural and religious events (i.e., music events, club festivals, Friday pray at the mosque, through the interviewers using their respective social networks). On the basis of a quota scheme, individuals were recruited from this participant pool for those age and gender strata that had insufficient numbers of participants (*n* = 96). In *Berlin,* the recruitment of participants (*N* = 604) took place through snowball sampling from the beginning of the data collection and through on-site surveys at public places (i.e., weekly markets, citizen registration offices, city projects, neighborhood initiatives, parent groups, a college, a mosque, etc.) in districts that had a high percentage and density of individuals with Turkish citizenship.

The quota scheme applied to the snowball sampling in Hamburg and Berlin contained the variables gender (male/female), age (18–29/30–49/50–65 years) and education level (high/middle/low), based on micro-census data (personal communication with regional statistical office, 2012). In both research centers, migration-specific methodological procedures were implemented, such as conducting focus groups before starting the survey, including public media campaigns as well as known key persons and stakeholders within the Turkish community, developing a bilingual field team and using bilingual information (e.g. letter, flyer, consent forms) and interview material, as well as establishing a bilingual-staffed telephone hotline, etc. [35]. The core face-to-face interviews (CIDI) were conducted by trained and regularly supervised bilingual lay interviewers (*n* = 28, 22 female and 6 male) and took place in centrally located, community-based, discrete interview offices. The interviewing of relatives and friends was not allowed. Overall, 458 of the completed interviews (*n* = 662) were conducted in the Turkish language, based on the self-selected language preference (German or Turkish) of the subjects. The study participants received an incentive of 10 Euro for each hour of the interview in the form of a gift card in Hamburg. In Berlin, the participants received 10 Euro twice: once for the screening interview and another for the main interview. The average length of the CIDI interview was 117 min (range: 26 to 360 min), excluding the quality assessment (section x).

### Measures

#### Composite international diagnostic interview, DIA-X CIDI Version 2.8(TR)

A computerized version (DIA-X, Diagnostic Expert System Interview) of the Composite International Diagnostic Interview (CIDI) was used as the core instrument of the study. The CIDI assessed the presence of mental disorders based on criteria of the DSM-IVTR [37] and ICD-10 compatible codes [38] to determine the lifetime-, 12 month-, and point prevalence (4 weeks) using a standardized decision tree algorithm [39]. This version of the CIDI is a further development of the Munich Composite International Diagnostic Interview (DIA-X/Munich-CIDI), which is based on the principle of Computer-Assisted Personal Interviewing (CAPI) and makes the standardized implementation of a clinically structured interview in a face-to-face setting possible [39, 40]. The psychometric quality criteria, in respect to objectivity, reliability and validity, have proved to be good [39–41]. Thus, the retest reliability of the DIA-X CIDI was good, and high concordance with DSM-IV diagnoses was found in a random population sample (*N* = 60), which was measured twice (interval: 38 days); the results ranged from kappa = 0.56 for any eating disorder to kappa = 0.81 for any anxiety disorder [40]. Good concordances were also found for diagnostic validity [41]. In general, the concordances between the clinician and CIDI diagnoses varied from kappa = 0.63 (any panic disorder) to kappa = 0.96 (any depressive episode). However, lower (although still acceptable) concordances were found for dysthymia (kappa = 0.54) and somatoform disorders (kappa = 0.50). Thus far, there is no Turkish language translation of the DIA-X/CIDI. In the preparatory phase, the DIA-X CIDI 2.8 (TR) used in this study was translated by means of a multi-step process into Turkish based on the TRAPD team approach by Harkness (2008) [42]. An analysis of the translated Turkish DIA-X CIDI 2.8 (TR) conducted on the basis of qualitative and quantitative measures shows an equally good quality and feasibility compared with the German version. Detailed information on the individual steps of the translation and the quality assurance process can be found elsewhere [43].

#### Sociodemographic data

A number of additional psychometric self-evaluation instruments, which were available in both languages (German and Turkish), were administered. The relevant instrument for the purposes of this paper, in addition to the CIDI, is the questionnaire on sociodemographic data. This questionnaire includes 50 main questions that were developed on the basis of the first [44] and second German national health survey [45] and the micro-census of 2010 [46]. Thus, the following components were assessed: a) *household* (e.g. questions about the number of household members, age and gender of all household members, family relationships of the interviewees with the household members); b) *migration background* (e.g. questions about country of birth, nationality, residence permit status, length of residence of the interviewees as well as of their parents and grandparents); c) *language* (e.g. questions on mother tongue, current language(s) spoken at home, self-estimated Turkish and German language proficiency); d) *ethnic, religious and cultural affiliation* (e.g. questions regarding which ethnic, religious and cultural groups the interviewees belong to or feel affiliated with); e) *educational level* (e.g. questions about country and duration of education, educational and vocational qualifications); f) *employment and retirement* (e.g. questions about current employment status, types and time of work, types and length of unemployment or retirement, household income); g) *utilization of health care services* (e.g. questions about consulting practitioners and health care facilities in Germany and Turkey, reasons for consulting health care services and experienced barriers; help-seeking behavior and requirements in cases of psychological strains and symptoms).

#### Household income and socioeconomic status

The net equivalent household income was calculated according to the modified Organisation for Economic Cooperation and Development (OECD) equivalence scale, taking into account the size of the household and the age of its members [34]. A 1.0 weight was assigned to the head of the household, every additional household member received a weight of 0.5 and children up to 15 years of age received a weight of 0.3. The total monthly net income was divided by the sum of the household members’ weights to obtain the equivalent disposable household income [34]. In reference to Lampert et al. (2013) [47], three income groups were constructed (low: ≤921€, middle: 922€-1417€, high: ≥1418€). The net equivalent income construct is a relatively valid indicator for determining the socioeconomic status because it can be assumed that the income categories (low/middle/high) reflect academic and employment qualifications and employment status [47].

### Statistical analysis

The data were weighted according to gender (female/ male), age (18 to 34/ 35 to 49/ 50 to 65 years), and education (low/ middle/ high) based on micro-census data that were provided for residents of Hamburg and Berlin with Turkish migration backgrounds (personal communication with regional statistical office, 2012). To avoid underestimation of standard errors and overestimation of significance (due to specific recruitment effects) as well as to obtain prevalence rates that are representative of the underlying population, all analyses were conducted using a *complex sampling* command that adjusts for sampling effects using the weighting information described above. In the first step of analysis, differences between first and second generations in sociodemographic variables (gender, age, marital status, current partnership, education, socioeconomic status, employment, nationality, residence permit status, mother tongue, and language at home) were tested formally using Χ^2^-Tests. Odds with according 95% confidence intervals (CIs) are presented for each comparison to estimate the effect sizes. In a second set of analyses, differences in prevalence rates of specific diagnostic groups between the first and second generation were tested using the same procedure as in the first analysis. Finally, in a third set of analyses, differences in prevalence rates of specific diagnostic groups (dependent variables) between the first and second generations (independent factor) were estimated using logistic regression models controlling for relevant sociodemographic variables. The selection of sociodemographic variables that were entered as confounders in the logistic regression models was based on content validity as well as the correlations between potential confounders. All analyses were conducted using SPSS (Statistic and Analysis Software) version 21.

## Results

Because the generation status (first vs second generation) was not available for 9 individuals, the following results are based on a total sample size of *n* = 653.

### Sociodemographic characteristics among first and second generation individuals with Turkish migration backgrounds

The distributions of the sociodemographic characteristics of the first and second generation are presented in Table 1. The first generation comprised the largest portion of the sample, at 84.5% (unweighted). According to the weighted percentage values, the gender distribution was nearly identical between the first and second generation, with 60% (women) to 40% (men). In contrast, significant differences were evident between the first and second generation pertaining to (1) age *p* < 0.001, (2) relationship status (marital status *p* < 0.001, current relationship *p* < 0.001), (3) socioeconomic aspects (educational level *p* = 0.001, socioeconomic status *p* = 0.034), (4) migration-related aspects (nationality *p* < 0.001, residence permit status *p* = 0.017) and (5) language aspects (mother tongue *p* < 0.001, language at home *p* = 0.001).

**Table 1:** Sociodemographic characteristics of individuals with Turkish migration backgrounds, weighted by gender, age and educational level

	First Generation (*N* = 502) not-weighted				Second Generation (*N* = 151) not-weighted				Total (*N* = 653)								
	N	Nw	%w	SE	N	Nw	%w	SE	N	Nw	%w	SE	95%		Test	(df)	*p*
Gender															X^2^ = 0.127	(1)	0.777
Women	292	332.154	62.3	5.6	88	74.802	60.5	5.1	380	406.955	62.0	5	50.1	72.5			
Men	210	201.160	37.7	5.6	63	48.736	39.5	5.1	273	249.896	38.0	5	27.5	49.9			
Age															X^2^ = 147.966	(1.499)	<0.001
18–29	51	42.534	7.7	2	86	52.742	51.3	5.2	137	95.276	14.5	2.1	10.1	20.3			
30–49	314	331.759	59.9	2.4	65	50.148	48.7	5.2	379	381.907	58.2	2.4	52.2	63.9			
50–65	137	179.552	32.4	1.2	**	**	**	**	137	179.552	27.3	1.1	24.6	30.2			
Marital status															X^2^ = 160.805	(2.029)	<0.001
Married	347	375.915	70.4	5.7	51	41.196	33.5	5.2	398	417.111	63.5	4.7	53.2	72.7			
Separated	30	30.895	5.8	1.9	5	3.755	3.1	1.9	35	34.65	5.3	1.6	2.8	9.8			
Divorced	72	77.706	14.5	3.2	14	13.406	10.9	2.1	86	91.112	13.9	2.3	9.8	19.3			
Widowed	12	13.555	2.5	0.8	1	0.267	0.2	0.2	13	13.822	2.1	0.6	1.1	3.9			
Never married	41	36.109	6.8	1.2	80	64.227	52.3	5	121	100.336	15.3	1.9	11.6	19.8			
Current partnership															X^2^ = 67.013	(1)	<0.001
Yes	348	373.336	69.9	5.6	51	36.98	30.1	4.1	399	410.316	62.5	4.9	51.8	72.0			
No	154	160.844	30.1	5.6	100	85.87	69.9	4.1	254	246.714	37.5	4.9	28.0	48.2			
Educational level ^*a*^															X^2^ = 55.802	(1.083)	0.001
Low	234	226.483	46.5	3.1	22	22.831	13.8	1.1	256	249.315	38.2	2.8	31.5	45.4			
Medium	105	103.652	21.3	1.4	51	56.387	34.1	7.1	156	160.039	24.5	2.4	19.2	30.7			
High	163	157.299	32.3	4.1	78	86.137	52.1	8.1	241	243.437	37.3	4.8	26.5	49.5			
Socioeconomic status															X^2^ = 18.488	(1.795)	0.034
Low (≤ 921)	284	326.743	72.4	5.7	75	71.271	66.7	5.6	359	398.014	71.3	4.5	61.0	79.8			
Medium (922–1417)	95	98.177	21.8	5	21	15.92	14.9	4.4	116	114.096	20.4	3.8	13.5	29.7			
High (≥ 1418)	48	26.358	5.8	1.7	26	19.603	18.4	4.8	74	45.961	8.2	1.9	5.1	13.2			
Employment															X^2^ = 6.147	(1.701)	0.261
Yes	230	230.038	43.1	6.5	71	58.02	47.2	5.9	301	288.058	43.8	5.9	32.1	56.3			
No	195	211.356	39.6	5.2	73	54.595	44.4	7.6	268	265.952	40.5	4.9	30.7	51.1			
Housewife/−man	77 ^*b*^	92.786	17.4	3.1	7	10.235	8.3	3.8	84	103.021	15.7	2.6	10.9	22.0			
Nationality															X^2^ = 70.276	(2.148)	<0.001
German	100	90.651	17.0	2.5	83	64.753	52.7	4.4	183	155.404	23.7	2.6	18.6	29.6			
Turkish	363	404.670	75.8	2.7	52	51.694	42.1	4.4	415	456.364	69.5	2.8	63.4	75.0			
Both	37	36.792	6.9	1.3	15	6.141	5	1.1	52	42.933	6.5	1.1	4.6	9.2			
Other	1	1.473	0.3	0.3	1	0.262	0.2	0.2	2	1.734	0.3	0.2	0	1.6			
Age at time of migration																	
< 13	118	145.420	27.9	3.7	**	**	**	**	118	145.420	27.9	3.7	20.8	36.4			
13–17	118	147.650	28.3	2.3	**	**	**	**	118	147.650	28.3	2.3	23.8	33.4			
18–25	148	137.700	26.4	3.1	**	**	**	**	148	137.700	26.4	3.1	20.5	33.3			
> 25	103	90.231	17.3	1.4	**	**	**	**	103	90.231	17.3	1.4	14.6	20.4			
Years in Germany																	
≤ 10	59	51.434	9.9	1.7	**	**	**	**	59	51.434	9.9	1.7	6.9	14.0			
11–20	124	132.916	25.5	1.5	**	**	**	**	124	132.916	25.5	1.5	22.4	28.8			
21–30	102	105.836	20.3	2.2	**	**	**	**	102	105.836	20.3	2.2	16.1	25.3			
≥ 30	202	230.815	44.3	2.0	**	**	**	**	202	230.815	44.3	2.0	40.2	48.5			
Residence permit status															X^2^ = 12.892	(1)	0.017
Limited	96	104.488	24.4	3.7	3	2.976	4.4	2.9	99	107.464	21.6	3	16.0	28.6			
Unlimited	290	324.105	75.6	3.7	70	65.412	95.6	2.9	360	389.517	78.4	3	71.4	84.0			
Mother tongue															X^2^ = 82.785	(2.811)	<0.001
German	1	0.684	0.1	0.1	16	12.851	10.5	4	17	13.535	2.1	0.9	0.8	5			
Turkish	434	445.800	83.8	2.6	99	83.946	68.3	4.4	533	529.747	80.9	2.8	74.3	86.2			
Both	27	37.151	7	2	35	25.259	20.6	3.6	62	62.41	9.5	2	6.1	14.5			
Other ^*c*^	38	48.318	9.1	2.2	1	0.794	0.6	0.6	39	49.111	7.5	1.9	4.4	12.5			
Language at home															X^2^ = 36.621	(2.424)	0.001
German	53	48.684	9.3	1.1	46	32.971	27	4.6	99	81.656	12.6	1.7	9.5	16.5			
Turkish	346	366.189	69.7	2.7	57	57.557	47.2	6.2	403	423.746	65.5	2.8	59.3	71.2			
Both	90	100.963	19.2	2.3	47	31.537	25.8	4.1	137	132.500	20.5	1.9	16.8	24.7			
Other ^*c*^	7	9.278	1.8	0.9	**	**	**	**	7	9.278	1.4	0.7	0.5	4.0			

Note: *w* weighted, *N* sample size, *SE* standard deviation, *CI* confidence interval, *df* degrees of freedom, *p* probability; ^a^ Educational level: high (Abitur = high school graduation, Fachhochschulabschluss = college of higher education graduation), middle (Realschulabschluss = middle school graduation), low (Hauptschulabschluss = secondary school graduation/degree/attempt; no degree). The Turkish graduations or degrees were transferred into these categories: high (Abitur = high school graduation, Fachhochschulabschluss = college of higher education graduation), middle (Ortaokul = middle school), low (primary school, no degree); ^b^This group contains one man; ^c^predominantly Kurdish or Zazaish

### Lifetime rates of DSM-IV mental disorders among first and second generation individuals with Turkish migration backgrounds

The estimated DSM-IV prevalence and comorbidity rates of mental disorders by generation among individuals with Turkish migration backgrounds are presented in Table 2. Overall, the results showed no significant differences between the first and second generation. However, the presence of any bipolar disorder did show a significant difference (*p* = 0.009), namely, the second generation was more likely to be affected than the first (36% vs 21%, respectively). Major depressive disorders showed a slight tendency towards a difference (*p* = 0.055), with a prevalence nearly twice as high in the first generation (15%) as in the second generation (7%).

**Table 2:** Lifetime prevalence rates of DSM-IV-TR disorders among individuals with Turkish migration backgrounds, weighted by age, gender and education level

	First Generation (*N* = 502) not-weighted			Second Generation (*N* = 151) not-weighted			Total (*N* = 653) not-weighted									
	n	%w	SE	n	%w	SE	N	%w	SE	95%	OR	(95%)	CI	Test	(df)	p
Mood Disorder																
Unipolar depression	154	32.9	2.6	37	26.6	3.6	191	31.7	2.1	27.5 - 36.2	0.740	0.451	1.219	X^2^ = 1.805	(1)	0.221
Any bipolar disorder	98	20.9	2.4	49	35.6	5	147	23.7	2.2	19.3 - 28.6	2.109	1.234	3.606	X^2^ = 11.924	(1)	0.009
Major depressive disorder	75	14.9	2.2	20	7.4	2.5	95	13.5	1.9	10 - 18	0.453	0.199	1.03	X^2^ = 4.806	(1)	0.055
Dysthymia (with hierarchy)	79	18	1.7	17	19.2	2.2	96	18.2	1.4	15.4 - 21.4	1.095	0.754	1.592	X^2^ = 0.103	(1)	0.655
Any mood disorder	197	42.9	3	58	37.7	5.3	255	41.9	2.2	37.3 - 46.7	0.805	0.433	1.498	X^2^ = 1.106	(1)	0.474
Anxiety Disorder																
Panic disorder	57	13.2	1.7	14	11.7	4.2	71	12.9	2.0	9.3 - 17.6	0.883	0.451	1.729	X^2^ = 0.199	(1)	0.674
Agoraphobia	103	21.6	1.9	22	16	5	125	20.5	1.7	17.1 - 24.4	0.699	0.301	1.624	X^2^ = 1.862	(1)	0.374
Social phobia	27	6	1.4	11	6.3	0.8	38	6.1	1.1	4.1 - 8.8	1.041	0.569	1.907	X^2^ = 0.012	(1)	0.873
Generalized anxiety disorder	17	2.7	0.9	4	2	1.3	21	2.6	1	1.1 - 5.7	0.726	0.301	1.748	X^2^ = 0.203	(1)	0.460
Specific phobias	98	19.7	2.3	27	22.1	1.8	125	20.2	1.9	16.5 - 24.4	1.164	0.776	1.745	X^2^ = 0.349	(1)	0.458
Obsessive compulsive disorder	47	9.2	1.6	14	8.9	2.2	61	9.1	1.1	7 - 11.7	0.993	0.419	2.355	X^2^ = 0.006	(1)	0.946
Any anxiety disorder	172	35.6	2	52	36.5	5.3	224	35.7	1.9	31.9 - 39.8	1.040	0.617	1.756	X^2^ = 0.034	(1)	0.880
Post-traumatic stress disorder	96	19.7	3.8	35	18.8	3.6	131	19.5	3.2	13.7 - 27	0.950	0.47	1.919	X^2^ = 0.054	(1)	0.859
Eating Disorder																
Anorexia nervosa	23	4.8	1.1	6	3.4	1.9	29	4.5	0.9	2.9 - 7	0.687	0.166	2.835	X^2^ = 0.470	(1)	0.588
Bulimia nervosa	10	2.1	0.6	2	0.8	0.5	12	1.8	0.5	1.1 - 3.2	0.351	0.065	1.886	X^2^ = 0.997	(1)	0.191
Any eating disorder	31	6.3	0.8	8	4.1	2.1	39	5.9	0.7	4.6 - 7.5	0.638	0.185	2.199	X^2^ = 0.830	(1)	0.458
Substance use disorder																
Alcohol abuse	48	7.6	0.7	15	7.8	3.1	63	7.6	0.9	6 - 9.7	1.030	0.423	2.509	X^2^ = 0.006	(1)	0.945
Alcohol dependence	14	2.9	0.5	4	2.6	1.6	18	2.9	0.5	2 - 4	0.883	0.213	3.653	X^2^ = 0.036	(1)	0.862
Medication abuse	8	1.6	0.7	2	2.9	2.2	10	1.9	0.8	0.8 - 4.5	1.775	0.372	8.469	X^2^ = 0.840	(1)	0.441
Medication dependence	5	0.7	0.5	1	1.7	1.8	6	0.9	0.7	0.2 - 4.7	2.397	0.853	6.736	X^2^ = 1.085	(1)	0.082
Nicotine dependence	203	41.4	2.7	40	29.7	6.1	243	39.2	2.3	34.6 - 44.1	0.596	0.292	1.216	X^2^ = 5.684	(1)	0.144
Any substance use disorder	224	44.4	2.3	48	34.3	7.4	272	42.5	2	38.4 - 46.7	0.654	0.299	1.432	X^2^ = 4.071	(1)	0.270
Any substance use disorder (without nicotine dependence)	55	9.2	0.7	18	12.2	3.8	73	9.8	1	7.9 - 12	1.379	0.654	2.908	X^2^ = 1.059	(1)	0.374
Somatoform disorder																
Pain disorder	128	26.9	3.6	39	27.8	5.1	167	27.1	3.4	20.5 - 34.8	1.051	0.62	1.782	X^2^ = 0.041	(1)	0.859
SSI4,6 (Undifferenzierte somatoforme Störung)	67	12.9	1.8	11	8.2	2.7	78	12	1.6	9 - 15.9	0.609	0.278	1.336	X^2^ = 2.104	(1)	0.183
Any somatoform disorder or syndrome	167	34.1	3.7	43	31.9	5.7	210	33.7	3.4	27 - 41.2	0.906	0.504	1.63	X^2^ = 0.218	(1)	0.724
Any mental disorder	398	80.4	1.5	112	72.1	7.6	510	78.8	1.7	75 - 82.2	0.631	0.269	1.484	X^2^ = 4.018	(1)	0.274
Any mental disorder excluding nicotine dependence and psychotic disorder	349	71.1	1.7	109	69.3	5.8	458	70.7	1.3	67.9 - 73.4	0.915	0.47	1.78	X^2^ = 0.145	(1)	0.796
Comorbidity																
One additional diagnosis	113	21	2.7	37	24.2	5.5	150	21.6	2.2	17.3 - 26.6	1.198	0.558	2.57	X^2^ = 0.611	(1)	0.616
Two additional diagnoses	90	20	2.1	21	10.6	2.7	111	18.2	1.4	15.5 - 21.3	0.472	0.21	1.062	X^2^ = 5.854	(1)	0.065
Three additional diagnoses	66	14	2	27	12	2.6	93	13.6	1.8	10.3 - 17.9	0.827	0.483	1.414	X^2^ = 0.363	(1)	0.481
Four additional diagnoses	49	9.7	1	16	11.4	5.5	65	10	1.2	7.8 - 12.8	1.199	0.365	3.943	X^2^ = 0.345	(1)	0.744
Five and more additional diagnoses	31	6.4	0.9	8	11.1	5.1	39	7.3	1.2	5 - 10.3	1.893	0.618	5.799	X^2^ = 3.285	(1)	0.263

Note: *w* weighted, *N* sample size, *SE* standard deviation, *CI* confidence interval, *OR* odds ratio, *df* degrees of freedom, *p* probability; Binge eating (which is normally included in any eating disorder) was removed because there were no cases of this diagnosis; Odds ratio: 1 = no differences in Odds, >1 = higher odds of the first generation, <1 = lower odds of the first generation.

Otherwise, the prevalence rates of the various disorder groups, and thus the total prevalence rates, were found to be similar in the first and second generations. This finding also applied to the comorbidity rates, which showed no significant differences between generations. One or more comorbid mental disorders was found to be present in around a fifth of the first generation. This rate decreased for the presence of more than three comorbid disorders (14%) and fell below 10% for the presence of four or five more comorbid disorders. In the second generation, only 24% were found to have more than one mental disorder. The prevalence rates for the presence of more than three and four or five comorbid mental disorders varied between 10.6% and 11.4%, respectively.

### Sociodemographic correlates of major diagnostic groups

Possible sociodemographic predictors of the five main diagnosis groups are presented in Table 3. Based on the correlation calculations, not all sociodemographic variables presented in Table 1 were included. Instead, the focus was placed on including variables with the highest likelihood of being independent of one another (*r* = 0.42). Significant effects were found in the following variables: female gender, older age and lack of current partnership, which were associated with any mood disorder. Furthermore, female gender was also associated with the four other disorder groups (any anxiety disorder, any eating disorder, any substance use disorder, any somatoform disorder/syndrome), and older age showed an additional significant effect related to ‘any somatoform disorder/syndrome’. Language at home was associated with ‘any eating disorder’ at a low level.

**Table 3:** Sociodemographic unweighted correlates of major diagnostic groups, lifetime disorders (*N* = 543)

	Any mood disorder				Any anxiety disorder				Any eating disorder				Any substance use disorder				Any somatoform disorder/syndrome			
	OR	CI	(95%)	p	OR	CI	(95%)	p	OR	CI	(95%)	p	OR	CI	(95%)	p	OR	CI	(95%)	p
Male gender = yes	0.528	0.364	0.767	0.001	0.409	0.277	0.603	<0.001	0.273	0.109	0.687	0.006	5.714	3.011	10.84	<0.001	0.421	0.283	0.625	<0.001
Age (continuous)	1.023	1.005	1.042	0.015	1.012	0.993	1.031	0.215	0.987	0.952	1.023	0.467	1.029	0.999	1.060	0.061	1.032	1.012	1.052	0.001
Current partnership = no	2.021	1.378	2.963	<0.001	1.128	0.766	1.661	0.542	1.506	0.715	3.169	0.281	1.555	0.846	2.855	0.155	0.948	0.638	1.409	0.790
Educational level low = yes	1.322	0.890	1.962	0.167	1.111	0.743	1.659	0.608	1.288	0.590	2.808	0.525	0.998	0.528	1.886	0.996	1.135	0.755	1.706	0.544
Socioeconomic status low = yes	1.118	0.754	1.657	0.580	1.419	0.945	2.129	0.091	0.568	0.264	1.223	0.148	0.869	0.475	1.590	0.649	0.949	0.632	1.426	0.802
Nationality Turkish = yes	0.883	0.577	1.351	0.567	0.891	0.578	1.373	0.601	2.337	0.835	6.542	0.106	0.919	0.475	1.782	0.804	0.983	0.633	1.528	0.940
First generation = yes	0.892	0.527	1508	0.670	0.827	0.485	1.412	0.487	0.776	0.275	2.191	0.632	0.836	0.357	1.957	0.679	0.674	0.390	1.166	0.158
Language at home Turkish = yes	0.821	0.555	1.212	0.320	0.935	0.628	1.393	0.743	2.546	1.032	6.282	0.043	0.788	0.430	1.444	0.440	0.712	0.475	1.067	0.099

Note: Multivariate linear regression analysis; because of the limited number of cases (see Table 1), the variables were combined as follows: gender (1 = men / 2 = women); age = continuous; current partnership (0 = no / 1 = yes); educational level (1 = low / 2 = medium + high); socioeconomic status (1 = low / 2 = medium + high); nationality Turkish (1 = yes / 2 = no); generation (1 = first-generation / 2 = second-generation); language at home Turkish (1 = yes / 2 = no). Any mood disorder: Nagelgerkes R^2^ = 0.086; any anxiety disorder: Nagelgerkes R^2^ = 0.076; any eating disorder: Nagelgerkes R^2^ = 0.097; any substance use disorder: Nagelgerkes R^2^ = 0.147; any somatoform disorder/syndrome: Nagelgerkes R^2^ = 0.082.

## Discussion

### Key findings

#### Differences between the first and second generation

The weighted sociodemographic characteristics (see Table 1) showed significant differences between the first and second generation, with the exception of gender and employment. Thus, the first generation was significantly more likely to be older, married/in a stable relationship, and to have a low educational level and was less likely to be in a high socioeconomic status. However, it should be noted that the generations had nearly identically low socioeconomic statuses. The first generation also showed significantly higher values for migration-related factors (i.e., Turkish nationality and limited residence permit status) and linguistic factors (i.e., mother tongue exclusively Turkish and language at home exclusively Turkish) than the second generation. Despite these significant group differences between the first and second generation, for almost all sociodemographic characteristics, no significant differences existed in the weighted lifetime prevalence rates (see Table 2), with the exception of any bipolar disorder (i.e., the second generation showed a higher prevalence) and potentially major depressive disorder (i.e., the first generation showed a higher prevalence). At this point, it is not possible to presume why only these two disorders showed differences, even contradictory, between the first and second generation.

Overall, the presented results do not confirm the epidemiological studies in the US, which indicated a higher lifetime prevalence of psychological disorders in the second generation compared to the first generation [14–20]. A frequently used explanation model is the *immigrant paradox*, which postulates (similar to the healthy-migrant effect) that foreign nativity and a continuing connection to the culture of origin has a protective impact on the development of physical and mental lifetime disorders [48]. Assimilation of the following generations is considered a risk factor for less optimal health, behavioral and education outcomes in children and adolescents [49]. However, studies investigating the concept of the immigrant paradox have presented conflicting findings, and thus no generally valid statements can be made [48, 49].

#### Overall lifetime prevalence

Considering all of the investigated disorders in the presented study, the lifetime prevalences are found to be very high; in a population-based German health survey from 1998 to 1999, the general German population showed a lifetime prevalence of mental disorders of 43% [50], whereas the presented study indicated a prevalence rate nearly twice as high, at 79%. However, the German health survey (1998/1999) provided no comparative concerning any anxiety disorder because of methodological difficulties [51]. Meanwhile, the Robert Koch Institute conducted a second German national health survey from 2008 to 2011 using the same computerized CIDI version as the presented study. Because there are currently no detailed publications presenting lifetime data, a detailed comparison between the German host society and the population group of individuals with Turkish migration backgrounds is not possible at this time. However, one publication offers some information on the lifetime data from the second German national health survey regarding depression [52]. Based on this, the lifetime prevalence of CIDI-diagnosed depression was 12% for the general German population. In comparison, the present study showed a lifetime prevalence of any mood disorder of 42%. These initial findings support the hypothesis that individuals with Turkish migration backgrounds in Germany are more likely to suffer from a psychological disorder at any time in life than the general population. However, as mentioned above, US surveys have found the opposite trend, i.e., migrant groups having lower prevalence rates in comparison to their host society [14–16]. The findings of the present study are more consistent with European surveys. This conclusion appears to be evident, as it can be assumed that there are more similarities in migration, historical and sociopolitical developments between neighboring European countries than between Germany and the USA. Nevertheless, it should be noted that the majority of the European non-clinical studies were conducted using self-report questionnaires, which predominantly examined only individual disorders in relation to point, 1 month or 12-month prevalence [24–27, 29]. Therefore, no differentiated and reliable comparison is possible with the presented data. However, when considering the larger epidemiological European studies, it becomes clear that the prevalence rates of mood disorders among migrants are higher than those of non-migrants [24, 28] and the German general population [52]. For example, Aichberger et al., (2010) [24], reported prevalence rates (measured on the Euro-D scale) of approximately 31% for the lifetime prevalence of depression in elderly European migrants (age ≥ 55 years). De Wit et al. (2008) [28], whose data were based on a clinically structured interview (CIDI 2.1), reported that Turkish migrants showed a lifetime prevalence of 31% for depression (vs 25% for Dutch non-migrants) and 15% for anxiety disorders (vs 12% for Dutch non-migrants). The comparability of these findings with the presented data is, however, limited because de Wit et al. (2008) [28] considered only a few subgroups of depression and anxiety disorders. Nevertheless, these reported prevalence rates of depression and anxiety were also very high; accordingly, it can be assumed that the presented data provided a realistic representation of the lifetime prevalence of mental disorders in individuals with Turkish migration backgrounds living in Germany or that the overestimations in lifetime data were limited.

Interestingly, and in contrast with the findings mentioned above from the US studies [15, 17, 18], there was one disorder group with an estimated 10% lifetime prevalence that was not more prevalent than in the 1998/1999 German national health survey and in fact had a nearly similar prevalence [50]: any substance use disorder (excluding nicotine). In contrast, a survey of pupils in a major German city showed differences in substance use between Turkish and German adolescents, in which the surveyed Turkish adolescents (1) used cannabis and alcohol less frequently, (2) assessed these substances as being more dangerous and (3) were less likely to consume cannabis or alcohol, even when given the opportunity [53]. A significantly lower alcohol consumption was also reported in adolescents following the Islamic religion [54]. Although this applied to 89% of those surveyed in the present study, the lifetime prevalence of any substance use disorder showed no difference with the German general population.

Concerning the lifetime prevalence of comorbidity, no comparisons with the German general population can be made at this time because there are no publications available to the best of our knowledge. However, based on the high lifetime prevalence of the presented disorder groups, it can be assumed that comorbidity is also higher in the population in the presented study than in the German general population.

#### Sociodemographic correlates

The risk of suffering from depression was around twice as high for women and singles. With increasing age, this risk increased by 1.023 (per year). These correlations were in line with findings for the German general population [50, 52] and for individuals with Turkish migration backgrounds [28, 29, 55]. In relation to the other disorder groups, the data only showed an increased risk for women. Age showed a significant association with any somatoform disorder/syndrome, which presumably corresponds to a general decrease in physical and mental health with increasing age or to a process of convergence with the health status of the host society as a result of acculturation [56]. In contrast to previous international and national findings, no significant correlations were found between the major disorder groups and (1) sociodemographic factors, namely, low educational level and low socioeconomic status due to unemployment and poverty [25, 26, 29, 57–59] nor with (2) migration-related factors, i.e., nationality, generation and language at home, as possible parameters of acculturation [55, 60, 61]. Why language at home showed a correlation with the occurrence of any eating disorder remains unclear.

### Strengths and limitations

The present paper provides important epidemiological data on the lifetime prevalence of mental disorders in the largest group with a migration background in Germany. Many efforts were made to reach the target population and to increase participation in the study. For these purposes, trust building and motivating measures were conducted (e.g., contacting popular key persons and stakeholders in the Turkish community, implementing public media campaigns, conducting on-site assessments, establishing a telephone hotline and centrally located, community-based survey offices, and offering incentives). A further strength of the study was the culturally sensitive survey methods: all information and survey materials were bilingually prepared. The core instrument of the survey (DIA-X CIDI Version 2.8) was translated and pre-tested in an elaborate, multistep process [43], and the bilingual interviewers were intensively trained and regularly supervised during the data collection.

Despite these efforts, some limitations can be assumed. The snowball sampling technique in Berlin and the additional snowball sampling in Hamburg, as well as the heterogeneous recruitment methods in the research centers, could have led to potential selection effects and overestimations of the presented lifetime prevalence data. Although a variety of daily life locations and cultural events were chosen during the snowball sampling, selection effects and limitations regarding the representativeness can be expected. Thus, it is possible that individuals who were already mentally burdened were more likely to participate in the survey, presumably because they had less fear of discussing the topic of mental strain or perhaps because they were hoping for a supportive conversation. In this context and as a possible explanation for the very low response rate of the study, a qualitative focus group investigation prior to the survey also indicated that the participants had strong prejudices and mistrust in research studies [62]. A further aspect might be the fear of lacking data protection and stigmatization within the Turkish community in the case of participation. The media campaign and the on-site assessments were unable to reach the trust-building effects, which had originally been intended and should be therefore readjusted in further research. Moreover, it seems conceivable that closer and long-term networking with the Turkish community might lead to greater willingness to participate in health research studies.

Further bias could also be due to an insufficient translation of the clinical, structured interview (DIA-X CIDI Version 2.8). After all, 69% of the participants conducted the interview in Turkish. However, an analysis of the translated DIA-X CIDI version showed a relatively good quality and feasibility compared with its German counterpart [43]. Nevertheless, the question can be raised of whether a cultural adaptation of the DIA-X CIDI could have resulted in a higher validity due to culture-specific subjective theories of disease and language-specific forms of expressing psychological symptoms [63]. In addition, the participants were asked to remember life stages and diagnoses that were potentially years or decades in the past. Because the majority of the participants were first generation migrants (84.5%), biases through memory gaps or distortions can be assumed.

Furthermore, the focus on two large cities/city districts with a high number and density of individuals with a Turkish migration background could have led to an overestimation of the prevalence rates. Consistent with this notion, increased lifetime prevalence rates in large cities have also been found for the general German population when compared to those in smaller towns and rural areas [52].

In addition to these methodological limitations and potential biases, the overall results of the study, which indicate high prevalence rates among first and second generation individuals with Turkish migration backgrounds, tend to confirm the migration-stress hypothesis. Thus far, evidence of a healthy migrant effect in individuals of Turkish migration backgrounds was predominantly found in relation to somatic findings, such as cardiovascular disease, cancer and mortality rates [64–66]. This seems evident because the target group of this study consisted of labor migrants (‘Gastarbeiter’) and their descendants. The first generation of this migrant group came to Germany in response to the German-Turkish recruitment agreement of 1961 [67]. They were required to undergo medical examinations and to be in good physical health to obtain working permission for their entry into Germany [68]. As Turkey’s political intention was to decrease their unemployment level through this labor recruitment agreement, the majority of the selected labor migrants came from rural and impoverished regions of Turkey [69]. In addition to the labor migrants, an additional migration group from Turkey consisted of refugees, who fled to Germany due to political persecution [69]. Therefore, it can be assumed that due to the burdening push factors in the country of origin (i.e., lower socioeconomic status due to low educational level, unemployment and poverty and political persecution), the main portion of the target group (first generation) in this study were already at a psychological disadvantage at the time of their migration. A 1996 study in Turkey showed much lower levels of lifetime mental disorders, for example, 7.4% for any anxiety disorder and 7.3% for any mood disorder. The comorbidity rates were also significantly lower than in the presented study. In addition to these potentially pre-existing burdening factors and experiences of stress as a result of the migration itself, further stressors in the post-migration phase, as well as for the second generation, can be presumed, such as experiencing discrimination, which has been reported to be more frequent in individuals with Turkish migration backgrounds than in other migrant groups in Germany [55, 70, 71]. There is evidence that perceived discrimination and minority status negatively affect mental and physical health [59, 71]. Perceived discrimination and social disadvantages were not explicitly investigated in this study. However, the data pertaining to socioeconomic status (low = 71%) indicated less optimal social conditions and opportunities for first and second generation individuals with Turkish migration backgrounds.

## Conclusion

The presented study indicates very high lifetime prevalence rates of mental disorders in individuals with Turkish migration backgrounds in Germany. A significant difference between the first and second generation could not be determined. Significant sociodemographic correlates (i.e., female gender, older age and no current partnership) were particularly present for the occurrence of any mood disorder. Female gender was also correlated with the other investigated disorder groups (i.e., any anxiety disorder, any eating disorder, any substance use disorder, any somatoform disorder/syndrome) and was a predictor of the development of a lifetime disorder. Despite the discussed potential selection effects and biases concerning the representativeness of the high lifetime prevalence, these findings provide initial, important, non-clinical data on the largest migrant group in Germany. However, the data were based on a cross-sectional survey design. To consider progressions in mental health and potential influencing factors, longitudinal studies should be implemented in further research. For this purpose, it seems advisable to consider the reasons and circumstances for the migration, the acculturation processes, and the potential influencing stress and protective factors. Regarding the clinical implications, the results showed a high need for appropriate prevention and treatment options for individuals with Turkish backgrounds including culture- and language-sensitive health care treatment and for facilitating access to the German psychosocial healthcare system.

## Abbreviations

CAPI: Computer-assisted personal interviewing
CIDI: Composite international diagnostic interview
CIs: Confidence intervals
DIA-X: Diagnostic expert system interview
DSM: Diagnostic and statistical manual of mental disorders
ICD: International classification of diseases
Munich-CIDI: Munich composite international diagnostic interview
OECD: Organisation for economic cooperation and development
SPSS: Statistic and analysis software
TR: Turkish
